# Practice Matters: Pro-environmental Motivations and Diet-Related Impact Vary With Meditation Experience

**DOI:** 10.3389/fpsyg.2020.584353

**Published:** 2020-12-16

**Authors:** Ute B. Thiermann, William R. Sheate, Ans Vercammen

**Affiliations:** ^1^Centre for Environmental Policy, Imperial College London, London, United Kingdom; ^2^Center for Mindfulness and Compassion, Cambridge Health Alliance, Cambridge, MA, United States

**Keywords:** mindfulness, compassion, connectedness with nature, sustainability, pro-environmental behavior, animal-protein consumption, motivation, self-determination theory

## Abstract

Mindfulness has emerged as a potential motivator for sustainable lifestyles, yet few studies provide insight into the relationship between mindfulness practice levels and individual engagement in pro-environmental behaviors. We also lack information about the significance of meditators’ behavioral differences in terms of their measurable environmental impact and the motivational processes underlying these differences in pro-environmental performance. We classified 300 individuals in three groups with varying meditation experience and compared their pro-environmental motivations and levels of animal protein consumption. Exceeding prior attempts to compare high-impact behaviors of mindfulness practitioners and non-practitioners, we created the most detailed classification of practice engagement by assessing frequency, experience and type of meditation practice. This nuanced view on mindfulness practice reveals that advanced meditators, who reported high levels of connectedness with nature (CWN), subjective happiness and dispositional mindfulness showed significantly more concern for the environment. They also demonstrated the lowest levels of greenhouse gas emissions, land occupation and water use related to their animal-protein consumption. This study is the first to follow a self-determination theory perspective to deepen our understanding of the motivational differences between meditator groups. We revealed that advanced meditators reported significantly more integrated motivation toward the environment than non-meditators. We also provided preliminary evidence for a new theoretical framework suggesting that experiential strategies such as mindfulness practices could strengthen the relational pathway of pro-environmental behaviors. Using sequential mediation analysis, we confirmed that the negative effect of mindful compassion practice on greenhouse gas emissions from animal-protein consumption is partially mediated by CWN and integrated motivation toward the environment. While our study does not support assumptions of causality, it shows that much can be learned by studying the motivations of advanced meditators for maintaining high levels of pro-environmental behavior.

## Introduction

The environmental crisis is accelerating, with climate change being one of the main drivers for environmental change and biodiversity loss. This creates negative impacts on ecosystem services and human well-being (Costello et al., 2009; IPCC, 2014; Díaz et al., 2019). Paradoxically, even though climate change has become increasingly tangible to the lay person, public opinions on climate change have changed little over the last decade (Egan and Mullin, 2017; Steentjes et al., 2017). The lack of individuals’ recognition of the gravity of the crisis stands in stark contrast with the need for individual level contributions to environmental conservation (Creutzig et al., 2016). Oftentimes neglected in the discussion on mitigation strategies, consumption levels must be reduced by a factor of five to attain the 2-degree target of global warming (Girod et al., 2013). This implies structural behavior change in areas like transport and diets, particularly the reduction of animal-protein consumption (Hedenus et al., 2014). A sustainable food transformation is indispensable not only to reach the targets agreed in the Paris Agreement, but also the sustainable development goals (Lucas and Horton, 2019). Changing people’s dietary preferences is a challenge that amounts to a socio-cultural revolution (O’Riordan and Stoll-Kleemann, 2015; Macdiarmid et al., 2016) and potential strategies to increase the willingness to adopting sustainable diets continue underexplored (Hartmann and Siegrist, 2017).

In the search for ways to promote sustainable lifestyles in Western populations, mindfulness is receiving growing attention (Ericson et al., 2014; Fischer et al., 2017). Thirty years of pioneer research showed that mindfulness is associated with environmental awareness and pro-environmental behaviors (PEB) (Thiermann and Sheate, 2020b). Some argue that “the promotion of mindfulness and loving-kindness meditation in schools, workplaces, and elsewhere could be construed as a policy that pays a ‘double dividend’ in that it could contribute both to more sustainable ways of life and to greater well-being” (Ericson et al., 2014, p. 78). Yet to date, causality has not been proven and there is limited information on *measurable* environmental savings related to mindfulness *practice*. With data obtained from an online survey of 300 adults in the United Kingdom, we investigated how different levels of meditation experience are related to animal-protein intake. We also tested core assumptions of the theoretical framework for this study, the two-pathway model of PEB (Thiermann and Sheate, 2020a).

### Background

#### Research Gaps in Mindfulness and Sustainability

The role of mindfulness in sustainability is a novel line of scientific inquiry and several research gaps are yet to be addressed. Mindfulness is a universal human capacity defined as “the awareness that arises when we intentionally pay attention in a kind, open discerning way” (Shapiro et al., 2018, p. 1694). While cross-sectional studies support the association between mindfulness and sustainable behavior, the existence of a causal relationship between the two concepts is still debated and will require a considerable amount of time and financial resources for longitudinal studies (Geiger et al., 2019b; Thiermann and Sheate, 2020b). This study focuses on two other crucial research gaps highlighted in Thiermann and Sheate (2020b).

First, from the several ways of conceptualizing and measuring mindfulness, most researchers rely on dispositional (also: trait) mindfulness to study correlations. Dispositional mindfulness is an individual’s capacity to bring mindful awareness to everyday life and increases over time with the practice of meditation (Kiken et al., 2015; Shapiro et al., 2018). Several researchers found a weak to moderate association between dispositional mindfulness and PEB (Geiger et al., 2019b), however, a dominant critique of this research is that only a few studies included meditation practitioners in their samples or even assessed practice parameters (Fischer et al., 2017; Thiermann and Sheate, 2020b). In order to determine if the implementation of mindfulness programs could promote sustainable lifestyles, it is imperative to understand the association between practice experience and PEB. Only a few studies have examined differences in PEB outcomes as predicted by the existence of an active mindfulness practice (Jacob et al., 2009; Panno et al., 2018; Loy and Reese, 2019). Also, it is important to clarify and distinguish between different mindfulness practices, because of their great variation in the underlying neuro-cognitive mechanisms and transformational potential. A recent empirical investigation by Matko and Sedlmeier (2019) identified a total of 309 commonly practiced meditation techniques. They clustered the most popular 20 into seven types, which differ in their degree of body orientation and how much physical movement they involve. Regarding the cognitive mechanisms of different meditation types, most meditations can be classified as belonging to three families: attentional, constructive and deconstructive meditations (Dahl et al., 2015). Most secular mindfulness-based interventions are primarily situated in the attentional family of practices that range somewhere along the continuum between developing focused attention and open monitoring skills (Vago and Silbersweig, 2012; Chiesa, 2013). Another type of practices that is gaining increasing attention in mindfulness research are meditations from the constructive family, particularly those with a relation and affect orientation such as loving-kindness and compassion meditations, also known as ethical enhancement practices (Vago and Silbersweig, 2012; Dahl et al., 2015). In a rigorous large-scale trial that studied the effect of three groups of practices in a “presence,” “affect” and “perspective” module, researchers found that affect-oriented practices such as loving-kindness and compassion meditation were most effective in promoting pro-social behaviors and altruistic tendencies (Böckler et al., 2018; Singer and Engert, 2019).

The second research gap is the lack of insight in measurable environmental impact. The measurement of PEB, defined as “behavior that harms the environment as little as possible, or even benefits the environment” (Steg and Vlek, 2009, p. 309), is a challenging task (Gatersleben et al., 2002; Kormos and Gifford, 2014; Gatersleben, 2018; Lange and Dewitte, 2019). Most mindfulness studies revert to behavioral antecedents and unvalidated self-report scales to determine strength of PEB. The Mindful Climate Action program is the only program designed to evaluate behavioral effects via environmental impact indicators such as greenhouse gas (GHG) emissions from diet, transportation and household energy (Grabow et al., 2018). However, results are limited to a pilot and feasibility study, and the effects from mindfulness practice would be difficult to extricate as the intervention combines mindfulness with environmental education modules (Barrett et al., 2016).

#### Can Mindfulness Promote Sustainable Diets?

Regarding individual behaviors, our diets are considered under the most impactful. Data from the Global Calculator show that if by 2050 everyone globally ate a healthy diet as recommended by the World Health, the world could save up to 15 gigatons of CO_2_ equivalents provided that the newly available land becomes reforested or used to grow bioenergy crops. These savings in GHG emissions amount to approximately one third of the world’s emissions in 2011 (Department of Energy and Cimate Change, 2015). This is reflected by a growing number of publications studying the relation between mindfulness and sustainable eating (Jacob et al., 2009; Fung et al., 2016; Böhme et al., 2018; Geiger et al., 2019a; Hunecke and Richter, 2019; Stanszus et al., 2019; Werner et al., 2020).

Fung et al. (2016) proposed a theoretical model where mindful eating is expected to improve the “awareness of the relationships between food and body, feelings, mind and interconnectedness between humans and the environment” (Fung et al., 2016, p. 1084), postulating that mindfulness should help maintaining both personal and planetary health. Stanszus et al. (2019) suggest a theoretical link between mindfulness and sustainable eating based on the potential of mindfulness to disrupt routines, promote physical and psychological well-being, strengthen values, pro-sociality and compassion as well as to improve the congruence between attitudes and behavior. Their research is based on an 8-weeks program combining mindfulness and environmental education (Fritzsche et al., 2018). The training proved effective in promoting mindful eating and changing antecedents of sustainable behavior, such as environmental attitudes and subjective well-being, but none of the intervention brought significant changes in sustainable eating behavior (Böhme et al., 2018; Geiger et al., 2019a; Stanszus et al., 2019).

Four correlational studies shed a more positive light on the relationship between mindfulness and sustainable eating. In a study with more than 800 mindfulness practitioners, Jacob et al. (2009) found a small but significant correlation between the frequency of mindfulness meditation and sustainable diets. Other studies showed that increased levels of dispositional mindfulness, particularly the ability to observe inner and outer experiences, correlated positively with more sustainable food consumption patterns (Hunecke and Richter, 2019; Richter and Hunecke, 2020). A study combining dispositional mindfulness and spirituality found that a supportive mindset marked by self-compassion and an earthly sense of spirituality were positively associated with increased PEB and more sustainable food choices (Werner et al., 2020).

In summary, these studies suggest that changes in sustainable eating and other PEB potentially develop with mindfulness practice over time (Mason et al., 2016; Geiger et al., 2019b). Furthermore, the relationship between mindfulness and sustainable behaviors seems to be indirect and mediated by a variety of factors such as sustainability values and beliefs, connectedness with nature (CWN), spirituality, subjective well-being, health awareness and emotional self-control (Jacob et al., 2009; Barbaro and Pickett, 2016; Aspy and Proeve, 2017; Park and Dhandra, 2017; Geiger et al., 2018, 2019b; Hunecke and Richter, 2019; Werner et al., 2020).

### Theoretical Framework

**FIGURE 1:**
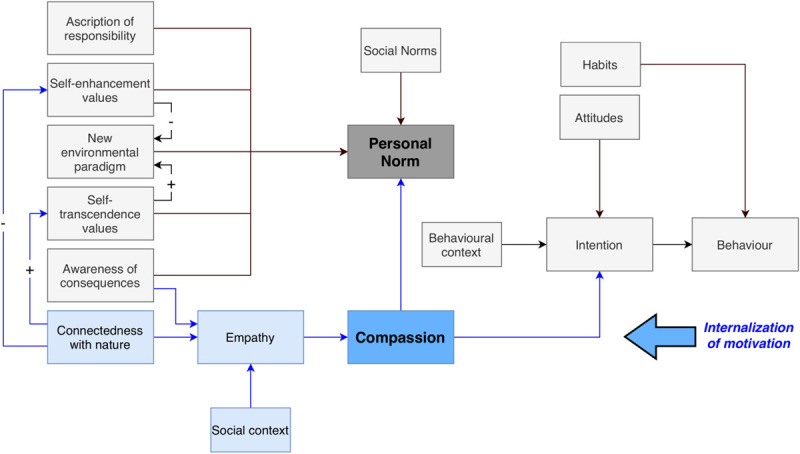
The 2-pathway model of PEB (Thiermann and Sheate, 2020a).

Theoretical studies establish six major arguments for the relationship between mindfulness and PEB: (1) increased awareness, (2) enhanced subjective well-being, (3) higher levels of CWN, (4) improved pro-social tendencies, (5) recognition of intrinsic values, and (6) openness to new experiences (Thiermann and Sheate, 2020b). Because these theoretical connections are not linkable to any of the prominent models explaining PEB (Steg and Nordlund, 2018), we proposed a 2-pathway model of PEB (see Figure 1) as an attempt to expand mainstream models of PEB and include mechanisms relevant to mindfulness (Thiermann and Sheate, 2020a). The greatest innovation provided by the model is the addition of the “relational pathway” of PEB, based on CWN, empathy and compassion as the driver of behavioral intention. This model suggests that with increased activation of the relational pathway through experiential strategies such as mindfulness, the motivation to act in favor of the environment becomes more internalized and self-determined, which ultimately improves behavioral outcomes and contributes to personal well-being.

The 2-pathway model of PEB is embedded in the wider framework of the self-determination theory and aims to overcome the widespread dichotomous understanding of environmental motivation as either extrinsic or intrinsic (Ryan and Deci, 2017; Thiermann and Sheate, 2020a). Kasser (2017) argues that self-determination theory, which gained reputation as the most well-researched theory on human motivation and well-being, might be best suited to explore the connection between PEB and individual well-being. Self-determination researchers found that both well-being and high-quality motivation for behaviors arise when an individual experiences satisfaction of inherent psychological needs for autonomy, competence, and relatedness. In application of the self-determination theory to the ecological context, Pelletier et al. (1998) describe six types of motivation toward the environment along a continuum: intrinsic, integrated and identified motivation are based on higher levels of need satisfaction and are therefore seen as the more self-determined motivation types and encourage higher quality and maintenance levels of PEB. Introjected, external and amotivation are the more controlled and less self-determined motivations which tend to collapse when a behavior involves effort, because the individual does not feel their inherent needs satisfied (Pelletier et al., 1998, 2011).

The 2-pathway model of PEB supports the assumption that experiential interventions such as mindfulness practice help to internalize environmental motivation because they help to intensify an individual’s relationship with nature and promote their needs for autonomy and competence. This might be particularly valid for meditators who formally cultivate compassion as part of their mindfulness practice (Thiermann and Sheate, 2020a). This shift toward more self-determined types of environmental motivation might further contribute to closing the attitude-behavior gap which hinders individuals from expressing their environmental ideals in action (Steg et al., 2015; Fischer et al., 2017; Kasser, 2017).

### Study Aim and Hypotheses

The aim of this study was twofold. First, we sought to address gaps in the mindfulness and sustainability literature by examining whether mindfulness practice (rather than dispositional mindfulness) relates to common predictors and measurable indicators of PEB, specifically the environmental impact generated by animal-protein consumption. The second goal was to provide preliminary evidence that mindfulness contributes to a shift in motivation toward the environment by activating the relational pathway of PEB. We focused on comparing the attitudes and behaviors of three groups with varying degrees of meditation experience. More specifically, we outline the following hypotheses:

H1: Dispositional mindfulness, subjective happiness and CWN progressively increase with the degree of meditation experience.

H2: Mindfulness practice is associated with a shift in the quality of motivation toward the environment which becomes more integrated and less amotivated.

H3: The level of self-reported environmental behaviors differs between practitioner groups, with advanced meditators showing the lowest environmental impact.

H4: CWN and integrated motivation toward the environment mediate the relationship between mindfulness practice and PEB.

## Materials and Methods

### Questionnaire Development

The survey was composed of psychometric scales, closed and open-ended questions. We used existing Likert-type scales with established psychometric properties to measure mindfulness, subjective happiness, motivation toward the environment and CWN. Additional items queried the presence and frequency of different forms of mindfulness practice and respondents’ dietary habits. We also obtained demographic details and invited general comments.

#### Psychometric Scales

Mindfulness was measured with the Comprehensive Inventory of Mindfulness Experiences (CHIME), currently the most comprehensive scale assessing mindfulness as a quasi-trait (Bergomi et al., 2013, p. 21). The authors recently revised the 37-item scale and provided an ordinal-to-interval conversion table, improving its validity and making it suitable for samples containing meditators and non-meditators (Medvedev et al., 2018).

We used the 4-item Subjective Happiness Scale (SHS) to assess self-reported wellbeing. The SHS does not specify the characteristics of happiness, thereby not forcing respondents to conform to either an eudaimonic or hedonic concept. The SHS has high internal consistency and excellent reliability (Lyubomirsky and Lepper, 1999; Lyubomirsky, 2020).

To capture motivations for PEB we used the Motivation Toward the Environment Scale (MTES; Pelletier et al., 1998). To distinguish an individual’s level of self-determination in PEB, the 24-item questionnaire rates the strength of their intrinsic, integrated, identified, introjected, external and amotivation toward the environment. Only respondents who had previously answered “Yes” to the question “Do you do things for the environment?” (93% of respondents) completed the MTES.

We used the Connectedness to Nature Scale (Mayer and Frantz, 2004) to measure trait levels of feeling connected with nature. The scale measures cognitive beliefs about one’s CWN (Perrin and Benassi, 2009), which has been demonstrated to be an important predictor of pro-environmental attitudes and behaviors (Pereira and Forster, 2015; Mackay and Schmitt, 2019; Whitburn et al., 2019).

Details of the psychometric scales are provided in Table 1.

**TABLE 1:** Overview of the psychometric scales included in the survey.

**Scale**	**Subscales**	**Response format**	**Example items**	**Cronbach’s α**
CHIME	Awareness toward internal experiences	6-point Likert scale	When my mood changes, I notice it right away	0.781
	Awareness toward external experiences		I notice details in nature, such as colors, shapes, and textures.	0.832
	Acting with awareness		I break or spill things because I am not paying attention or I am thinking of something else.	0.637
	Accepting and non-judgmental orientation		During both ups and downs of life, I am kind to myself.	0.859
	Decentering and non-reactivity		When I have distressing thoughts or images, I am able to feel calm soon afterward.	0.875
	Openness to experiences		I try to stay busy to avoid specific thoughts or feelings from coming to mind.	0.752
	Relativity of thoughts		It is clear to me that my evaluations of situations and people can easily change.	0.602
	Insightful understanding		In everyday life, I notice when my negative attitudes toward a situation make things worse.	0.771
MTES	Intrinsic motivation	7-point Likert scale	For the pleasure I experience while I am mastering new ways of helping the environment.	0.945
	Integrated motivation		Because taking care of the environment is an integral part of my life.	0.943
	Identified motivation		Because it is a reasonable thing to do to help the environment.	0.961
	Introjected motivation		I think I’d regret not doing something for the environment.	0.905
	External motivation		Because other people will be upset if I don’t.	0.887
	Amotivation		Honestly, I don’t know; I truly have the impression that I’m wasting my time doing things for the environment.	0.888
SHS	None	7-point Likert scale	Some people are generally very happy. They enjoy life regardless of what is going on, getting the most out of everything. To what extent does this characterization describe you?	0.918
CNS	None	5-point Likert scale	I often feel a sense of oneness with the natural world around me	0.890

#### Mindfulness Practice

Taking an inclusive approach in targeting mindfulness practitioners, we first asked respondents whether they practiced any form of mindfulness (yes/no). Those answering affirmatively were asked subsequent questions about years of experience in years and weekly practice frequency, regarding both moving meditation (e. g. yoga postures, tai chi, qui gong, other martial art formations) and non-moving meditation (e. g. focused attention to one or more elements, such as to one’s body, breath, conscious awareness, or to a particular word, thought or emotive state) separately. We distinguished between moving and non-moving meditations based on observations that in the West, many people practice yoga asanas and martial arts as a fitness discipline without cultivating a mindful orientation, and the degree of mindfulness applied to the practice varies greatly dependent on the discipline and teacher. Those who practice forms of moving meditation such as yoga with the specific intention to cultivate mindfulness typically include a form of non-moving meditation in their practice, e. g. at the end of a yoga class. As such, we strictly allocated respondents to meditator groups based on their experience in non-moving meditation to increase the likelihood that those classed as (advanced) meditators had indeed benefitted from the neurocognitive mechanisms of an on-going *intentional mindfulness* practice. **Advanced meditators** are those who had been practicing non-moving meditation for at least a year and who practiced at least 3–4 times a week. **Novice/infrequent practitioners** are those who had been practicing for less than 1e year and who practice just once or twice per week or less. In addition, because affect-oriented practices have been shown to support pro-sociality more effectively, we also asked respondents to indicate if they practiced “compassion” as part of their mindfulness practice or as a separate practice (yes/no).

#### Diet-Related Environmental Impact: GHG, Land Occupation, Water Use

Following previous studies, we measured animal-protein consumption by asking how often respondents ate meat, fish, eggs, and dairy (Scarborough et al., 2014). For each of the animal-proteins, respondents indicated weekly frequency. They also stated whether they were actively trying to reduce their consumption of animal-proteins. Those who answered “yes” stated their reasons by distributing 100 percentage points between the five categories “personal health,” “animal welfare,” “environment and climate change,” “weight control,” and “other.”

We modeled three environmental impact measures associated with respondents’ weekly animal-protein consumption: **GHG emissions, land occupation** and **water use.** The model is based on data from the UK-specific report *Eating For Two Degrees* (WWF, 2017). The report uses country averages of GHG emissions, water use and land occupation associated with 100g of the most common food products, considering national proportions of imported (vs.) local produce, conventional (vs.) organic origin and other variations in production. The impact values were generated in a life-cycle perspective from cradle-to-mouth, including raw materials, agriculture, transport, retail, packaging, waste and preparation of the food in the household. For a detailed explanation of the environmental impact indicator (see Supplementary Appendix A).

### Questionnaire Distribution

Ethics approval was granted by the Imperial College Research Ethics Committee (21st May 2018). We distributed the survey online via the Qualtrics platform between June and September 2018 and targeted a range of audiences in the United Kingdom to sample across a range of characteristics of interest. Mindfulness practitioners were invited in Facebook groups and via newsletters of London-based mindfulness networks. Respondents of different diet types were approached via Facebook groups such as Vegans United Kingdom, Vegans London, Vegetarians, BBQ and Grilling, CountryWoodSmoke United Kingdom, London Foodies, and health and fitness themes. To incentivize respondents, they could win one of 20 Amazon vouchers to the value of 10 GBP. Additionally, to cover more general population features, we acquired 99 responses from Mechanical Turk^1^; 9 were removed because the responses failed to meet basic quality criteria.

### General Data Analysis Strategy

We conducted an *a priori* power analysis using G^∗^Power 3.0.10 (Faul et al., 2007) to determine the desired sample size for detecting a medium sized difference between the practitioner groups regarding performance in PEB. This was based on previous findings by Panno et al. (2018) who compared PEB of two practitioner groups and reported an η^2^ effect size of 0.01 at *p* < 0.05 with 95% test power. The medium effect size was later confirmed in another study by Loy and Reese (2019). Our estimated sample size was *N* = 252.

The preliminary dataset of 430 registered responses was cleaned for subsequent data analysis using MATLAB software. We excluded respondents who failed to provide information on their dietary habits or their mindfulness experience (even if they completed other parts of the questionnaire). For any given psychometric scale with more than 10% of datapoints missing, respondents were excluded from further analysis on that scale. When less than 10% were missing, missing data points were substituted with the average of all response values for the respective scale or subscale. The final sample includes 300 respondents. The data analysis was executed in IBM SPSS Statistics version 26. Whenever possible, we controlled for gender effects or separated outcomes by gender, because different patterns in pro-environmental attitudes have been observed between men and women (Vicente-Molina et al., 2018).

For all MANCOVAs, we used Fisher’s LSD *post-hoc* tests for pairwise comparisons of practitioner groups. We tested for equality of covariance matrices using Box’s M test and we tested for equality of error variances using Levene’s test. Due to the assumption that MANCOVA is robust to violations for sample sizes over 30 we proceeded with the analysis even when Box’s M was significant (*p* < 0.001) (Allen and Bennett, 2008). When equality of error variances could not be assumed for all variables, we used the Games-Howell *post-hoc* test to identify differences between the groups. For the MANCOVAs where gender was included as a covariate, we used Sidak’s adjustment for multiple pairwise comparisons. Detailed descriptive statistics and correlations between the key dependent variables are reported in Supplementary Appendix B. Apart from the GHG, land and water use variables, which are highly correlated and therefore assessed in separate ANOVAs, other variables included in MANOVA analyses were moderately correlated.

## Results

### Sample Description

**FIGURE 2:**
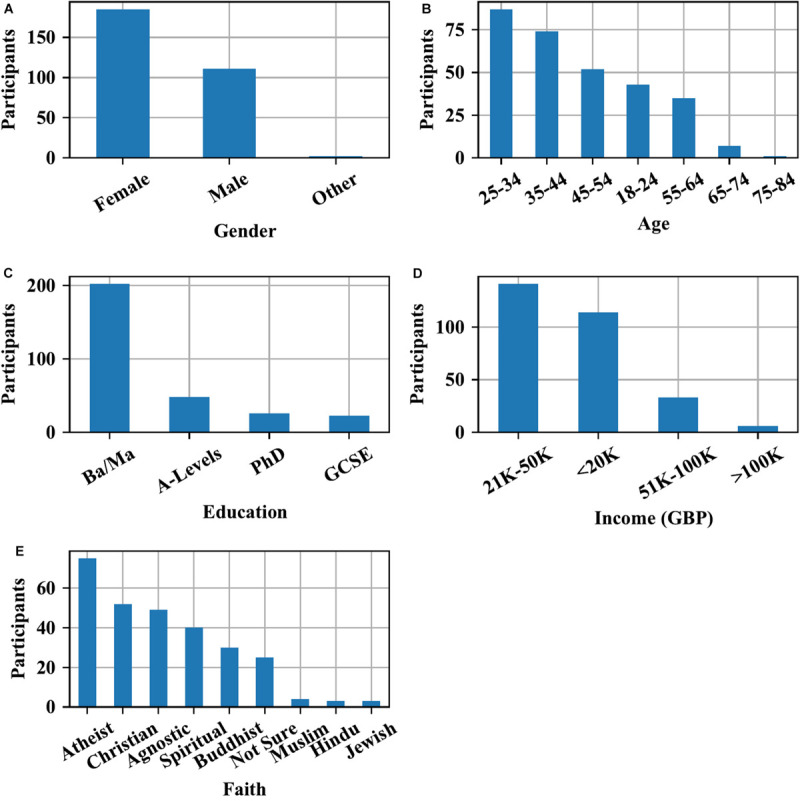
Distribution of sample characteristics in the sample of 300 respondents. **(A)** shows self-identified gender, **(B)** shows age at time of responding, **(C)** shows highest degree attained, **(D)** shows average income per year, **(E)** shows religious affiliation.

Descriptive statistics for gender, education, age, income and faith groups are provided in Figure 2.

In total, 186 (61.7%) respondents stated that they practice mindfulness (moving or non-moving), while 115 (38.3%) do not practice. In total, we classified 123 respondents (41%) as non-practitioners, 85 (28.3%) as infrequent/novice practitioners and 92 (30.7%) as advanced meditators.

Gender was unevenly distributed across the three groups, with males dominant in the non-practitioner group (63 men, 58 women) compared to the female-dominated infrequent/novice (23 men, 60 women) and advanced (25 men, 67 women) practitioner groups. 2 respondents chose “other” and 2 did not state gender.

With increasing meditation experience, respondents more frequently practiced compassion. In the non-meditator group, only 3 (1%) practiced compassion vs. 120 (40%) who did not practice. Of the infrequent/novice meditators, 47 (15.7%) practiced compassion whereas 38 (12.7%) did not. In the advanced group, 83 (27.7%) reported practicing compassion while 9 (3%) stated they did not.

Four diet types were represented in the sample of all practitioner groups, totaling 184 (61.3%) meat eaters, 22 (7.3%) pescatarians, 27 (9%) vegetarians and 64 (21.3%) vegans; 3 (1%) remained unknown. Table 2 summarizes the distribution of diet types per meditator group and gender.

**TABLE 2:** Distribution and percentages of diet types per meditator group and gender.

	**Non-meditator**				**Infrequent/novice meditator**				**Advanced meditator**			
	**Female**		**Male**		**Female**		**Male**		**Female**		**Male**	
	***n***	**%**	***n***	**%**	***n***	**%**	***n***	**%**	***n***	**%**	***n***	**%**
Meat eater	25	13.6	60	32.6	25	13.6	22	12	33	17.9	17	9.2
Pescatarian	2	9.1	1	4.5	5	22.7	0	0	11	50	3	13.6
Vegetarian	7	25.9	0	0	4	14.8	0	0	14	51.9	2	7.4
Vegan	23	35.9	2	3.1	25	39.1	1	1.6	9	14.1	3	4.7

*Percentages are attributed per row; unknown diet type and other gender are not listed.*

### H1: The Three Practitioner Levels Show Differences in Their Levels of Mindfulness, Subjective Happiness and Connectedness With Nature

We employed a multivariate general linear model (MANCOVA) on the mindfulness, happiness and CWN scale scores, using practice level as the independent variable and gender as a covariate. Dependent values were total scale scores on the CHIME, SHS and CWN. The scales and subscales were found to be internally consistent and alpha values are presented in Table 1.

The practitioner groups showed moderate differences on the three dependents, *F*(6, 574) = 16.793, *p* < 0.001); Wilk’s Λ = 0.724, η*_*p*_*^2^ = 0.149. Gender had a relatively small, but statistically significant effect on the dependents, *F*(3, 287) = 6.372, *p* < 0.001; Wilk’s Λ = 0.983, η*_*p*_*^2^ = 0.062. In follow-up univariate ANCOVAS we found medium-sized differences between practice levels regarding subjective happiness [*F*(2, 289) = 15.94, *p* < 0.001; η*_*p*_*^2^ = 0.099], small to medium differences on CWN [*F*(2, 289) = 11.80, *p* < 0.001; η*_*p*_*^2^ = 0.076] and large differences in dispositional mindfulness [*F*(2, 289) = 47.04, *p* < 0.001; η*_*p*_*^2^ = 0.246]. Women were more connected with nature than men, although the effect was relatively small [*F*(3, 289) = 12.91, *p* < 0.001; η*_*p*_*^2^ = 0.043], but did not significantly differ on subjective happiness [*F*(1, 289) = 0.16, *p* = 0.694; η*_*p*_*^2^ = 0.001] or dispositional mindfulness [*F*(1, 289) = 1.196, *p* = 0.275; η*_*p*_*^2^ = 0.004]. See pairwise comparisons between groups in Table 3.

**TABLE 3:** *Post-hoc* comparison of practitioner group differences on subjective happiness, CWN and dispositional mindfulness.

**Dispositional mindfulness**						
				**Sidak comparisons: *p*-value [CI for the difference]**		
**Meditation practice**	***n***	**Mean**	***SD***	**1**	**2**	**3**

1. Non-practitioner	119	3.66	0.46			
2. Infrequent/novice meditator	82	3.80	0.52	0.121 [−0.027, 0.337]	–	
3. Advanced meditator	92	4.33	0.58	<0.001 [0.512, 0.865]	<0.001 [0.346, 0.722]	–

**Subjective happiness**						

				**Fisher’s LSD (*p*-value) [CI for the difference]**		
**Meditation practice**	***n***	**Mean**	***SD***	**1**	**2**	**3**

1. Non-practitioner	119	3.71	1.49	–		
2. Infrequent/novice meditator	82	4.39	1.25	0.001 [0.274, 1.081]	–	
3. Advanced meditator	92	4.82	1.39	<0.001 [0.715, 1.496]	0.044 [0.011, 0.845]	–

**Connectedness with nature (CWN)**						

				**Fisher’s LSD (*p*-value) [CI for the difference]**		
**Meditation practice**	***n***	**Mean**	***SD***	**1**	**2**	**3**

1. Non-practitioner	119	3.57	0.71	–		
2. Infrequent/novice meditator	82	3.91	0.70	0.011 [0.058, 0.444]	–	
3. Advanced meditator	92	4.11	0.61	<0.001 [0.273, 0.646]	0.041 [0.009, 0.408]	–

### H2: Group Differences Manifest as a Shift in the Quality of Motivation Toward the Environment Which Becomes More Integrated and Less Amotivated

We conducted a multivariate analysis of variance (MANOVA) with practice level as the independent variable and all six motivation types (intrinsic, integrated, identified, introjected, external and amotivation) as outcome variables.

Of the 300 respondents, 279 people confirmed engagement in PEB and completed the MTES scale. Of those who declared no PEB, 61.9% are non-meditators, 28.6% are infrequent/novice meditators, and 9.5% are advanced meditators.

The groups differences between the motivations were medium-sized, *F*(12, 504) = 4.57, *p* < 0.001; Wilk’s Λ = 0.813, η*_*p*_*^2^ = 0.098. Follow-up univariate tests revealed that the groups showed small but significant differences in integrated motivation *F*(2, 257) = 6.49, *p* = 0.002; η*_*p*_*^2^ = 0.048, introjected motivation *F*(2, 257) = 3.06, *p* = 0.049; η*_*p*_*^2^ = 0.023 and amotivation *F*(2, 257) = 9.24, *p* > 0.001; η*_*p*_*^2^ = 0.067. They did not differ on the other motivational types intrinsic motivation *F*(2, 257) = 0.15, *p* = 0.863; η*_*p*_*^2^ = 0.001, identified motivation *F*(2, 257) = 0.10, *p* = 0.909; η*_*p*_*^2^ = 0.001 and external motivation *F*(2, 257) = 2.02, *p* = 0.135; η*_*p*_*^2^ = 0.015.

**FIGURE 3:**
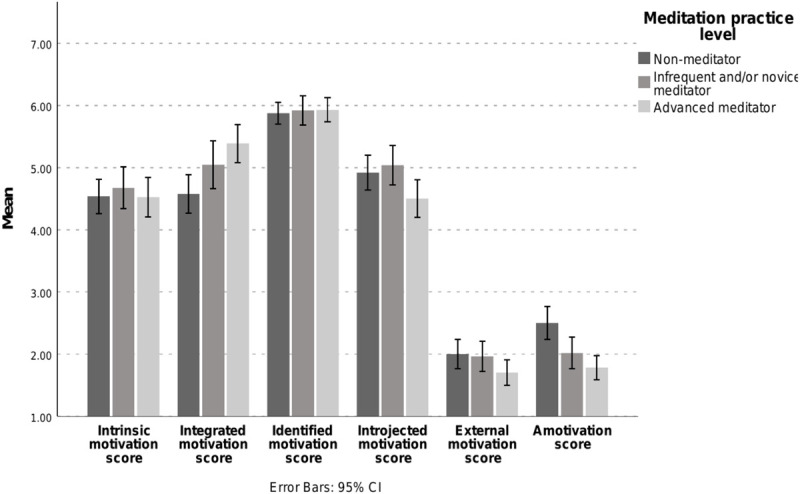
Comparison of the mean scores per meditation practitioner group for all six different motivation types. Note that only integrated motivation significantly differs between advanced meditators and non-meditators (*p* < 0.01). Amotivation significantly differs between advanced meditators and non-meditators (*p* < 0.001) and infrequent/novice meditators and non-meditators (*p* < 0.05).

Figure 3 shows the mean scores for the different motivations by practice group.

Significant pairwise differences are shown in Figure 3 and Table 4. Pairwise differences for the four motivation types without significant differences can be observed in Supplementary Appendix C.

**TABLE 4:** *Post-hoc* comparison of practitioner group differences on six different motivation type.

				**Games-Howell comparisons (*p*-value)**		
**Meditation practice**	***n***	**Mean**	***SD***	**1**	**2**	**3**
**Integrated motivation**						
1. Non-practitioner	102	4.56	1.59	–		
2. Infrequent/Novice practitioner	73	4.99	1.67	0.200 [−0.159, 1.014]	–	
3. Advanced meditator	81	5.39	1.39	0.001 [0.3089, 1.3439]	0.242 [−0.984, 0.186]	–
**Amotivation**						
1. Non-practitioner	102	2.49	1.35	–		
2. Infrequent/Novice practitioner	73	2.04	1.08	0.035 [0.026, 0.886]	–	
3. Advanced meditator	81	1.78	0.87	<0.001 [0.322, 1.096]	0.249 [−0.1213, 0.627]	–

### H3: The Level of Self-Reported Environmental Behaviors Differs Between the Groups, With Advanced Meditators Showing the Lowest Environmental Impact

We tested intentions to reduce or limit consumption of animal protein (yes/no) using binary logistic regression. Because dietary habits and intentions differed by gender, we repeated the analysis for men and women separately (Supplementary Appendix D). Within the subsample of “reducers,” we employed MANCOVA with the reported relative importance (scores) of four diet motivations (i.e., “personal health,” “animal welfare,” “environmental concerns,” and “weight control”) as outcome variables, practice level as the independent variable and gender as a covariate. To compare mean environmental impact metrics (GHG emission, land occupation and water use) of the three groups we used three separate univariate general linear models (ANCOVA), including gender as a covariate and following up the main effects with pairwise comparisons. Because of the high participation of vegans in the survey, disproportionally increasing their share in the less experienced practitioner groups, we repeated the analysis excluding all vegans (Supplementary Appendix D).

Of non-meditators, 61 (49.6%) stated their intention to reduce animal-protein consumption and 62 (50.4%) stated no such intention. The proportion of reducers increased for both infrequent/novice (60 reducers, 70.6%; 24 non-reducers, 28.2%; 1 unknown) and for advanced meditators (65 reducers, 70.7%; 27 non-reducers, 29.3%). The differences in proportions between the groups were statistically significant, χ^2^(4, *N* = 300) = 16.70, *p* = 0.002. Results from binary logistic regression showed that non-meditators were significantly less likely to reduce their animal-protein consumption in comparison to advanced meditators (Wald (1) = 9.43; *p* = 0.002; CI [0.231, 0.724]). The model predicts that the odds of not reducing animal-protein consumption were less than half [Exp(B) = 0.409] for advanced meditators than for non-meditators. In an additional analysis of the reduction effect by gender, we observed that the trend was mostly determined by the men in the sample, while women showed a generally high willingness to reduce animal-proteins across all practitioner groups (Supplementary Appendix D).

**FIGURE 4:**
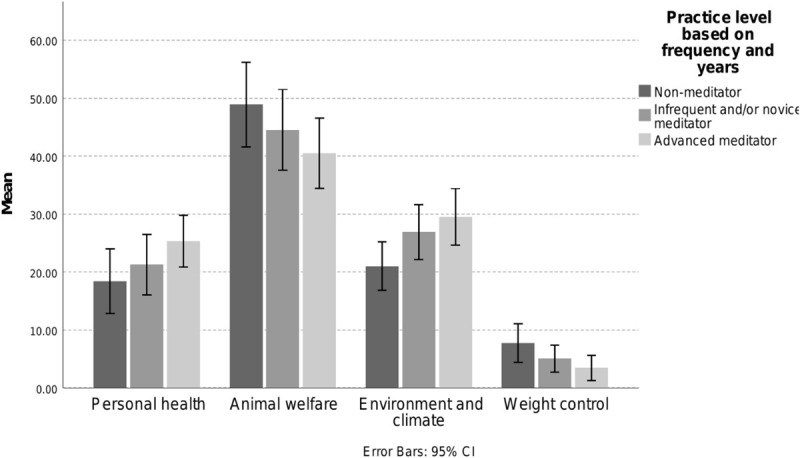
Comparison of the mean percentage points for the reasons for reducing animal-protein consumption, per meditator group. Note that only the scores for environmental and climate concern differ significantly between advanced meditators and non-meditators (*p* < 0.05).

One hundred and eighty five respondents who reported intentions to reduce animal-protein consumption also indicated their reasons for the reduction. Figure 4 shows the mean percentage points each of the practitioner group attributed to the different reasons for their reduction behavior.

Both practice level [*F*(8, 358) = 2.65, *p* = 0.008, η*_*p*_*^2^ = 0.056) and gender [*F*(4, 178) = 3.38, *p* = 0.011; η*_*p*_*^2^ = 0.071] had a significant small and small to medium sized effect on motivations for reducing meat consumption. Follow-up tests identified that small significant differences between practitioner levels were observed on the relative importance ascribed to environment and climate reasons [*F*(2, 181) = 3.566, *p* = 0.030; η*_*p*_*^2^ = 0.038], but not with respect to personal health [*F*(2, 181) = 1.71, *p* = 0.184; η*_*p*_*^2^ = 0.019], animal welfare [*F*(2, 181) = 1.472, *p* = 0.232; η*_*p*_*^2^ = 0.016] and weight control [*F*(2, 181) = 2.677, *p* = 0.072; η*_*p*_*^2^ = 0.029]. Men and women showed a small difference in terms of the relative importance ascribed to health reasons [*F*(1, 181) = 5.067, *p* = 0.026; η*_*p*_*^2^ = 0.027] and animal welfare [*F*(1, 181) = 5.510, *p* = 0.020; η*_*p*_*^2^ = 0.03], but not for environment/climate [*F*(1, 181) = 0.44, *p* = 0.438; η*_*p*_*^2^ = 0.002] or weight control *F*(1, 181) = 0.24, *p* = 0.628; η*_*p*_*^2^ = 0.001]. Pairwise comparisons between the practitioner groups are provided in Table 5. Compared to non-meditators, both novice/infrequent and advanced meditators assigned greater importance to the environment as a reason for reducing meat consumption, but the difference reached statistical significance only for advanced meditators.

**TABLE 5:** Multiple comparisons of practitioner group mean percentages of different reasons for reducing animal-proteins.

**Personal health**						
				**Sidak comparisons (*p*-value) [CI for differences]**		
**Meditation Practice**	***n***	**Mean**	***SD***	**1**	**2**	**3**

1. Non-practitioner	61	18.41	21.74	–		
2. Infrequent/novice meditator	59	21.22	20.43	0.655 [−12.597, 4.993]	–	
3. Advanced meditator	65	25.3	17.97	0.187 [−2.004, 15.059]	0.836 [−11.443, 5.992]	–

**Animal welfare**						

				**Fisher’s LSD (*p*-value) [CI for differences]**		
**Meditation Practice**	***n***	**Mean**	***SD***	**1**	**2**	**3**

1. Non-practitioner	61	48.92	28.54	–		
2. Infrequent/novice meditator	59	44.88	26.96	0.266 [−4.153, 14.972]	–	
3. Advanced meditator	65	40.51	24.47	0.094 [−1.358, 17.195]	0.602 [−6.970, 11.987]	–

**Environment and climate**						

				**Fisher’s LSD (*p*-value) [CI for differences]**		
**Meditation Practice**	***n***	**Mean**	***SD***	**1**	**2**	**3**

1. Non-practitioner	61	21.02	16.26	–		
2. Infrequent/novice meditator	59	26.95	18.49	0.094 [−0.968, 12.296]	–	
3. Advanced meditator	65	29.51	19.73	0.009 [2.154, 15.021]	0.381 [−3.651, 9.497]	–

**Weight control**						

				**Sidak comparisons (*p*-value) [CI for differences]**		
**Meditation Practice**	***n***	**Mean**	***SD***	**1**	**2**	**3**

1. Non-practitioner	61	7.72	13.02	–		
2. Infrequent/novice meditator	59	5.12	9.08	0.498 [−2.187, 7.066]	–	
3. Advanced meditator	65	3.46	8.66	0.065 [−0.188, 8.789]	0.699 [−2.726, 6.447]	–

We compared the mean environmental impact factors (GHG emissions, land occupation and water use) based on the groups’ reported consumption of animal-proteins for the duration of 1 month. We found small statistically significant differences between groups on GHG emissions, *F*(2, 290) = 4.051, *p* = 0.018; η*_*p*_*^2^ = 0.027. The covariate gender had a medium-sized impact on GHG, *F*(1, 290) = 44.48; *p* < 0.001; η*_*p*_*^2^ = 0.133. Similarly, there was a small significant main effect of practice level on land use impacts *F*(2, 290) = 3.860, *p* = 0.022; η*_*p*_*^2^ = 0.026 and a medium-sized effect of gender on land use impacts *F*(1, 290) = 42.963, *p* < 0.001; η*_*p*_*^2^ = 0.129. Finally, the groups showed a small significant difference in water use, *F*(2, 290) = 4.032, *p* = 0.019; η*_*p*_*^2^ = 0.027 and gender had a medium-sized effect on water use *F*(1, 290) = 44.589, *p* < 0.001; η*_*p*_*^2^ = 0.13. Table 6 compares the total mean values of all three environmental impact factors of the three groups for 1 month of eating animal-proteins. This includes pairwise comparisons of practitioner groups. The estimated marginal mean for the environmental impact indicators was significantly higher for non-practitioners over advanced meditators, and for non-practitioners over novice/infrequent meditators. The environmental impact indicators do not differ significantly between novice/infrequent and advanced meditators except when vegans were excluded from the analysis (Supplementary Appendix E).

**TABLE 6:** Comparison of total mean environmental impact (GHG emissions, land occupation, water use) per practitioner group for 1 month of eating animal-proteins, with gender as a covariate.

				**Fisher’s LSD (*p*-value) [CI for difference]**				**Yearly savings NP vs. AM**	
**Meditation practice**	***n***	**Mean Month**	**SD**	**1**	**2**	**3**	**Mean Year**		**Compares to**
**GHG EMISSIONS (KG CO_2_-EQ)**									
1. NP	120	47.31	36.42	–			567.7	215.52	Return flight from London to Frankfurt
2. IM	82	31.9	32.21	0.044 [0.222, 17.68]	–		382.8		
3. AM	92	29.35	26.41	0.007 [3.14, 20.04]	0.566 [−6.40, 11.67]	–	352.2		
LAND OCCUPATION (M^2^*A)									
1. NP	120	48.88	37.66	–			586.5	216.12	Area needed to keep 4 free-range hens on grass pasture, or 293 hens in a Sykes henyard (excluding production of 6t of straw per year for henyard)
2. IM	82	32.95	33.29	0.042 [0.34, 18.40]	–		395.4		
3. AM	92	30.87	27.05	0.010 [2.80, 20.28]	0.648 [−7.17, 11.51]	–	370.4		
**WATER USE (M^3^)**									
1. NP	120	0.39	0.30	–			4.68	1.8	12 days of UK average household water use for one individual
2. IM	82	0.26	0.27	0.041 [0.003, 0.147]	–		3.12		
3. AM	92	0.24	0.22	0.008 [0.025, 0.164]	0.607 [−0.055, 0.094	–	2.88		

*The three columns on the right compare the mean consumption per year and exemplify the reduction of environmental impact by the advanced meditators vs. non-practitioners. NP, non-practitioners; IM, Infrequent/novice meditators; AM, Advanced meditators (Department for Business Energy and Industrial Strategy, 2017; EnergySavingTrust, 2013; Plamondon, 2016).*

In Table 6 we also estimated the environmental impact of each group associated with eating animal-proteins for 1 year, comparing the savings of advanced meditator vs. non-meditators with an every-day example.

### H4: CWN and Integrated Motivation Toward the Environment Mediate the Relationship Between Mindful Compassion Practice and Environmental Behavior

**FIGURE 5:**
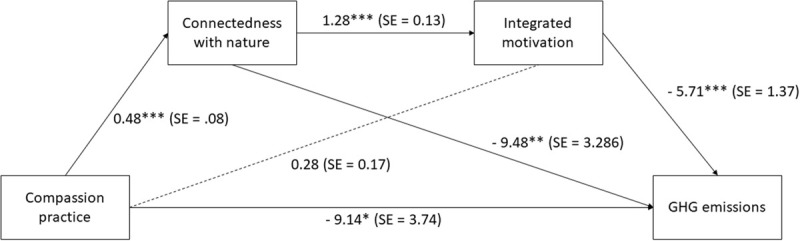
The sequential mediation effect of CWN and integrated motivation in the relationship between compassion practice and GHG emissions. ^∗∗∗^*p* < 0.001; ^∗∗^*p* < 0.01; ^∗^*p* < 0.05; all presented effects are unstandardized; standard errors are indicated in parenthesis; compassion practitioners coded as 2 and non-practitioners coded as 1.

We ran a sequential mediation analysis (model 6) using the PROCESS tool v3.4 by Andrew F. Hayes, with 10,000 bootstrap samples and a confidence interval of 95. We applied a binary predictor based on the existence of compassion practice (X), because affect-oriented mindfulness practices have been shown to provide the strongest impact on prosocial tendencies (see section “Research gaps in mindfulness and sustainability”). We assume that the relational pathway for PEB established by the 2-pathway model becomes stronger with the existence of such affect-oriented practices as part of respondents’ general mindfulness practice. Almost all the advanced practitioners in our sample reported practicing some form of compassion meditation, as well as a small group of infrequent/novice practitioners. Regarding the outcome variable to test our assumption, we chose to use GHG emissions (Y) as the representative for PEB performance. Repeating the analysis with the other two environmental impact variables would make these additional analyses redundant because the three variables are highly correlated (see Supplementary Appendix B). We analyzed CWN as the first mediator (M1) and integrated motivation as the second mediator (M2), as shown in Figure 5.

The total effect of the model was statistically significant, *B* = −18.78, *SE* = 3.8, *t* = −4.94, *p* < 0.001, 95% CI [−26.26, −11.3]. Compassion practice showed a significant positive effect on CWN, *B* = 0.48, *SE* = 0.08, *t* = 6.25, *p* < 0.001, 95% CI [0.33, 0.63], while its effect on integrated motivation was not significant, *B* = 0.28, *SE* = 0.17, *t* = 1.66, *p* = 0.09, 95% CI [−0.05, −0.6055]. This indicates that those who practice affect-oriented meditation practices such as compassion do show higher levels of CWN than non-practitioners. The practice of compassion also showed to be significantly negatively associated with GHG emissions, *B* = −9.14, *SE* = 3.74, *t* = −2.44, *p* = 0.02, 95% CI [−16.51, −1.7726], meaning that those who practice compassion meditation tend to emit less carbon from their animal-protein consumption than those who don’t. The direct effect of CWN on integrated motivation was significant and positive, *B* = 1.28, *SE* = 0.13, *t* = 10.28, *p* < 0.001, 95% CI [1.04, 1.53] and its effect on GHG significant and negative *B* = −9.49, *SE* = 3.29, *t* = −2.89, *p* = 0.004, 95% CI [−15.95, −3.02]. This means that those with higher levels of CWN do show higher integrated motivation toward the environment, and reduced carbon emissions from diet. Also, integrated motivation is negatively associated with GHG emissions: *B* = −5.71, *SE* = 1.37, *t* = −4.17, *p* < 0.001, 95% CI [−8.40, −3.01]. Regarding the indirect effects, we found that compassion practice was negatively associated with GHG emissions while partially mediated by CWN, *B* = −0.1307, *SE* = 0.06, 95% CI [−0.2647, −0.0387]. In comparison, the indirect effect of compassion practice on GHG through integrated motivation was not significant: *B* = −0.0488, *SE* = 0.03, 95% CI = [−0.12, 0.01]. In combination of both mediators, the indirect effect of compassion practice on GHG emissions through CWN and integrated motivation was significant and negative: *B* = −0.1084, *SE* = 0.3, 95% CI = [−1.79, −0.05]. This shows that the relational component of CWN plays a positive role in explaining the reduced GHG emissions of compassion meditators. However, the R-squared of the total effect was 0.084 indicating that our model only explained an estimated 8.4% of the variance in GHG emissions.

## Discussion

Mindfulness and its benefits develop through practice and accrue over time (Carmody and Baer, 2008; Bergomi et al., 2015; Franquesa et al., 2017). To gauge the potential of mindfulness interventions as a tool for promoting more sustainable lifestyles, we need a deeper understanding of how different levels of engagement with the practice translate into PEB and environmental impacts. The aim was to examine how one high-impact behavior, animal-protein consumption, is associated with mindfulness practice.

Corresponding with our hypotheses, we found that diet-related environmental impacts were lower for meditators compared to non-meditators, regardless of experience. Only advanced meditators showed the expected shift toward a more self-determined and strongly internalized motivation, where PEB become an integral part of a person’s self-concept. Advanced meditators also showed significantly more concern for the environment than non-practitioners. In line with the 2-pathway model of PEB, we confirmed that much of the mitigating effect of mindful compassion practice on PEB was mediated by relational aspects.

Regarding hypothesis I, meditators showed progressively higher subjective happiness and CWN compared to non-meditators, but only advanced meditators showed significantly elevated dispositional mindfulness. The latter observation is in line with previous research showing that continued meditation practice is crucial for developing and maintaining mindfulness (Bergomi et al., 2015). Yet in our sample, happiness and CWN showed to be greater even for those with limited mindfulness practice, somewhat moderating the assumption that the development of greater psychological well-being depends on the extent of engagement with the practice (Carmody and Baer, 2008). Our results also reflect experimental research by Aspy and Proeve (2017) who showed that even after a short meditation exercise, CWN increased significantly. Overall, our results indicate that mindfulness, subjective happiness and CWN might be interrelated and practice dependent (Howell et al., 2011).

For hypothesis II, we confirmed significantly higher levels of integrated motivation in the advanced meditator group compared to non-practitioners, and lower levels of amotivation in both the advanced and the infrequent/novice meditator group. All groups reported similar levels of intrinsic motivation toward the environment which might seem contradictory to research showing that mindfulness helps clarify intrinsic values (Ericson et al., 2014; Franquesa et al., 2017; Wamsler et al., 2017) and dampens the pursuit of extrinsic goals such as financial gain (Brown et al., 2009). Yet, we advocate an interpretation of our results that leaves behind the dichotomous understanding of environmental motivation that has been gaining attraction in mainstream environmental psychology (Thiermann and Sheate, 2020a). Self-determination theorists distinguish between *intrinsic motivation* and *self-determined extrinsic motivation* (such as integrated and identified motivation). Intrinsic motivation is “the innate tendency to engage in an activity for the sole pleasure and satisfaction derived from its practice. […] The behavior is an end in itself.” (Pelletier et al., 1998, p. 441). However, for behaviors that “do not occur spontaneously but are rather required by the social world” (Pelletier et al., 1998, p. 462), such as PEB, the aim is their successful internalization which occurs “when an instrumental behavior has been valorized to an extent such that it becomes part of the person’s self-definition” (Pelletier et al., 1998, p. 441). Even though PEB might emerge from external sources at the start, they can become fully self-determined (*as if* intrinsic) and an expression of how a person creates meaning. Hunecke and Richter (2019) showed that the relationship between mindfulness and sustainable diets is influenced by personal meaning-making processes. While mindfulness can be practiced with individually varying degrees of spirituality, most often it is directed at inner growth and human development as well as contemplating one’s intrinsic values and purpose in life (Garland et al., 2015). Werner et al. (2020) reported that natural spirituality, a concept connected to mindfulness practice, encourages greater responsibility for one’s actions and higher levels of intrinsic (to be understood as internalized) motivation. This trend also is reflected in the study of worldviews which found that individuals with more intrinsically oriented worldviews, especially those interested in inner growth, engage more frequently in pro-social behaviors and sustainable lifestyles (Hedlund-de Witt et al., 2014). It is therefore not surprising that the advanced meditators showed the highest levels of self-determined extrinsic motivation for PEB.

For hypothesis III, we did not only assess intentions and reasons for animal-protein reduction, but also compared the average environmental impacts from 1 month of eating animal-based products. Instead of employing dispositional mindfulness as the main predictor variable as in most studies of mindfulness and PEB, we built an indicator based on mindfulness practice frequency and years of experience. Only three other studies in the field used such practice indicators. Panno et al. (2018) and Loy and Reese (2019) applied a broad filter by determining the existence (yes/no) of any mindfulness practices. Our approach resembles Brown et al. (2009) who assessed at least the frequency of non-moving meditation practice. Our results mirror all three studies affirming that self-reported PEB (both the behavioral intention to reduce animal-proteins and the mean environmental impact) are enhanced for meditators compared to non-meditators, independent of the level of meditation experience. However, their underlying reasons differed: only advanced meditators demonstrated greater concern for the environment and climate as the reason for reducing consumption of animal protein, compared to non-meditators.

Hunecke and Richter (2019) found that mindfulness predicted more sustainable food consumption but not vegetarianism, opening questions about the importance of ecological vs. moral norms. Our results identify animal welfare as the leading reason for reducing meat consumption in all groups. Yet, advanced meditators cultivate a more holistic perspective on personal health, animal welfare and the environment. A possible explanation might be that a consistent meditation practice promotes a sense of interconnectedness with all beings and nature, allowing meditators to balance their individual needs with those of other beings and the environment (Hanley et al., 2017; Vieten et al., 2018). The relative prominence of personal health reasons in the advanced meditator group, even if not to a significant level, may explain some of their environmental savings because healthier diets also tend to be more sustainable (Van Dooren et al., 2014). This aligns with Geiger et al. (2018) who highlighted an indirect path from mindfulness to ecological behavior through health behaviors. Nevertheless, our results reinforce that environmental motivations play a significant role in the dietary decisions of advanced meditators.

Instead of assessing diet type as a binary indicator (Wamsler and Brink, 2018; Hunecke and Richter, 2019) or via Likert-scales (Stanszus et al., 2019; Werner et al., 2020), this study provides a more nuanced picture on diet-related environmental impact. As a result, we recorded several meat eaters with lower self-reported environmental impact than some vegetarians or pescatarians, because they generally ate animal-proteins with moderation. Our analysis further showed that the groups with at least some mindfulness experience had a significantly lower environmental impact than those without. To determine if the environmental savings are meaningful in practical terms, we compared the impact from a year worth of animal-protein consumption to daily-life examples of one United Kingdom citizen. To provide a sense of scale, such a saving extrapolated to every individual in the United Kingdom economy with 66.4 million inhabitants (Office for National Statistics, 2019) and a yearly GHG emission rate of 364.1 million tons (BEIS, 2019) results in a reduction of 14,319,148.8 tons^2^ of GHG emissions. This compares to a 3.8% reduction in the UK’s yearly emissions rate. Our study is the first to compare the environmental impact from the diets of different meditator groups which common Likert-type PEB scales fail to provide.

For hypothesis IV, the low R-squared value showed that several factors beyond mindfulness practice affect diet-related environmental impact. Yet, we could show that the weak relationship between mindful compassion practice and GHG emission reductions was partially mediated by relational factors, particularly by CWN but also a more internalized motivation for PEB. While previous studies have shown a reciprocal relationship between dispositional mindfulness and CWN (Schutte and Malouff, 2018), little evidence exists regarding the impact of the practice of mindfulness on CWN (Aspy and Proeve, 2017). Furthermore, our mediation analysis indicates that mindful compassion practice could be effective as an experiential strategy that strengthens the relational pathway of PEB and therefore deepens the motivation toward the environment (Thiermann and Sheate, 2020a). The importance of a compassionate mindset for sustainable diets also was discussed by Werner et al. (2020).

### Limitations

Creating and comparing three different groups of meditators based on practice frequency and experience is one of the most important innovations of this study, though it involves several limitations. There is no established guidance on how to distinguish advanced from infrequent/novice meditators. While we based our groupings on clear criteria, they represent artificial demarcations. For instance, the experience of advanced meditators varied greatly from 2 to over 15 years of practice. Many additional criteria could be used to classify advanced meditators, such as the total days spent on silent retreats, whether meditators use audio-guidance or meditate in silence, the average duration of meditation sessions, and whether the meditators seek out mindfulness teachings. Also, because of the large variability of moving meditation without scientific evaluation of their effect on trait mindfulness (such as vinyasa-style yoga, walking, or surfing), we refrained from integrating moving meditation as part of our categorization. This potentially disadvantaged some individuals who attain high levels of mindfulness through practices such as classical hatha yoga or qui gong.

Regarding the dietary impact indicator, the high-level focus on four types of animal products excludes other relevant factors of a sustainable nutrition such as the purchase of products from regional, seasonal, organic, fair-trade, and small-scale agriculture origin. Furthermore, within the animal-protein groups, we applied averages of several products which can largely differ in impact, e. g. beef steak and processed sausages. Because we generally attributed an impact of zero to vegans, there is no variation of the impact within this diet group.

Even though we asked detailed questions about the frequency of practice and consumption of animal-proteins, we expect that some social desirability response bias and recall bias is still represented in our data (Subar et al., 2015). The same applies to the psychometric scales, known to attract social desirability biases (Latkin et al., 2017). Respondents also might have been primed by the order of the questions and favored pro-environmental responses after engaging in the environmental scales. However, the effects would have affected all meditation groups equally and are therefore unlikely to have confounded the group effects.

We did not recruit a random or representative sample and as a result of our recruitment strategy, focusing on specific social groups and self-selection, the sample represents a disproportionally high percentage of vegans, particularly in the non-practitioner and infrequent/novice meditator groups which might have played in favor of the average environmental impact of these groups. The sample also includes a high proportion of university graduates and female respondents. Generally, because the survey was advertised as related to the topic of diets, well-being, environment and mindfulness, this likely attracted those who are altruistically inclined. The sampling bias therefore might also contribute to the small effect sizes in the analyses.

Finally, women were overrepresented in our sample and typically show stronger pro-environmental attitudes and behaviors (Lee et al., 2013; Kennedy and Kmec, 2018; Di Fabio and Rosen, 2019). While we have accounted for gender influences in our analysis and the general effect of practice remains significant, we suggest that future research on mindfulness and sustainable lifestyles should take an explicitly gendered approach. For example, our results suggest that at least for some behaviors (e.g., intention to reduce animal-proteins) women are engaging regardless of whether they meditate or not, while men are more likely to do so if they are also engaged in mindfulness practice.

## Conclusion

Our exploratory study showed that advanced meditators are happier, more deeply motivated toward the environment and they generate less environmental impact from animal-proteins. This makes mindfulness practitioners a prime target to learn more about individual ways to combine greater personal and planetary well-being. Yet the proof for causality remains an unresolved research gap in the area: is it the practice of mindfulness that renders people more interested in inner growth and sets them on a path to sustainability? Or are those who seek for meaning and self-improvement the ones drawn to mindfulness and therefore more likely to maintain a regular practice? Because our study showed that environmental motivations and ecological concern only significantly improved for advanced meditators with more than a year of practice (many meditate for more than 10 years) we suggest that future studies regarding causality accompany new meditators for a period of *at least* 1 year to detect changes in worldviews and lifestyles. At the same time, the longer the period of measurement, the more essential it is to account for other factors such as changes in social context or the engagement in Buddhist teachings. Qualitative research could greatly enhance this process.

With this study we strove to overcome the narrow research focus on dispositional mindfulness commonly applied in the field. This pioneer work did not only examine how mindfulness practices relate to different antecedent factors for PEB. It also is the first to quantify their effect on real world measures of environmental impact which provided tentative insights into the environmental benefits a widespread adoption of mindfulness practices could potentially entail. With this approach, we hope to spark future research ideas that focus on testing and operationalizing mindfulness programs as a policy tool for sustainable development.

## Data Availability Statement

The raw data supporting the conclusions of this article will be made available by the authors, without undue reservation. To receive the data, please contact the corresponding author: ute.thiermann15@imperial.ac.uk.

## Ethics Statement

The studies involving human participants were reviewed and approved by the Imperial College Research Ethics Committee https://www.imperial.ac.uk/research-ethics-committee/committ ees/icrec/. The patients/participants provided their written informed consent to participate in this study.

## Author Contributions

UT designed and executed this research as part of her doctoral thesis, including writing of this manuscript. AV supported the project as an advisor and particularly supported data analysis, and presentation and discussion of the results. WS was overseeing the work in his function as Ph.D. supervisor, and supported preparation of the manuscript and major discussion points. All authors contributed to the article and approved the submitted version.

## Conflict of Interest

The authors declare that the research was conducted in the absence of any commercial or financial relationships that could be construed as a potential conflict of interest.

## Footnotes

1 A crowdsourcing network: https://www.mturk.com/

2 Kg CO_2_eq value extracted from Table 6, converted to tonnes: 0.216. Then multiplied by 66,440,000 inhabitants.
