# Supplementary material for: Practice Matters: Pro-environmental Motivations and Diet-Related Impact Vary With Meditation Experience
Source: Front Psychol. 2020 Dec 16;11:584353. doi: 10.3389/fpsyg.2020.584353 (PMC7772404; doi:10.3389/fpsyg.2020.584353)
Supplement: Supplementary file 1 [file Data_Sheet_1.pdf]

Supplementary Material

1 APPENDIX A - Environmental Impact indicator calculations

Supplementary Material should be uploaded separately on submission. Please include any Based on the survey's statements of frequency, each of the respondents' environmental impact parameters (GHG emissions, land use, water use) were calculated per month in a stepwise approach:

1. **Create average impact values for food groups**: The WWF database contains impact values for a long list of products in each of the food groups meat, fish, eggs and dairy. In order to determine the impact associated with 100g units from these broader food groups, we used the average impact of all food products per food group. For example, the meat related GHG emissions are based on the averaged emissions for processed meat, beef, lamb, pork and poultry.

2. **Calculate monthly consumption per individual**: The WWF report found that in average, adults in the UK eat 92g/day of meat, 26g/day of fish, 196g/day of dairy and 8g/day of eggs (WWF, 2017, p. 46). These daily consumption values are multiplied by the self-reported frequency individuals stated to consume products from each food group, per month. The resulting values in grams are converted to units of 100g to match the WWF database, e. g. 392g of meat equals 3.92 units of 100g.

3. **Calculate monthly environmental impact per individual**: The individual monthly consumption values for each food group were finally multiplied with the previously calculated average values for GHG emissions, land use and water use per 100g of food.

The calculated values for the environmental impact refer exclusively to the consumption of animal-proteins and do not evaluate the overall sustainability of the individuals' complete diet. Factors such as personal preferences for local (vs) imported produce or for organic (vs) conventional agriculture have not been assessed on an individual level. Even though this might reduce the complexity and accuracy of the individual impact indicator, this decision is justified by publications advocating that large parts of the impact in the three categories GHG emissions, land-use and water use are associated to animal products and the production of feed for livestock (Harwatt, 2018; Springmann et al., 2018). A shift towards a more plant-based diet as recommended by national guidelines or seen in Mediterranean, pesco-vegetarian, vegetarian and vegan diets would not only improve the sustainability score but also the health score of the average diet (Behrens et al., 2017; Van Dooren, Marinussen, Blonk, Aiking, & Vellinga, 2014). However, a study examining the GHG emission values associated with different diet types in the UK finds that a complete vegan diet in average still accounts for 1055kg $CO2$ per year, compared to 2624kg $CO2$ per year for an omnivorous diet with high meat consumption (Scarborough et al., 2014).

To test the plausibility of our assumptions we compared the range of individual GHG emission values in our sample with the UK specific emission values associated with adult diets published by Scarborough et al. (2014). We compared their emission data related to individual meat consumption by subtracting the emissions associated with "fish-eaters" from both the "high meat-eaters" and the "low meat-eaters" diet scenarios (Scarborough et al., 2014, p. 185). The data published by Scarborough

et al. (2014) show that in the high meat scenario, approximately 1186kg CO2eq per year are emitted from meat alone while in the low meat scenario, the proportion reduces to 266.45kg CO2eq. By our indicator, a person with a high meat intake, eating meat five to seven times per week, scores 842kg CO2eq per year from meat only, versus a person eating meat only sporadically who emits approximately 70kg CO2eq per year from meat. This narrower range of emissions captured by our indicator most likely is due to the restricted number of self-report options which we averaged to monthly frequencies of eating meat on 0 days (never), 2 days (sporadically,

less than 4 times per month), 6 days (once or twice a week), 14 days (three to four times a week, every second day), and 24 days (five to seven times a week, daily) which provide a ceiling effect at the high end.

While our indicator reduces detail due to the five response categories provided in the questionnaire, it still allows for a higher level of accuracy in comparing individuals than simply requesting their diet type. For example, some meat eaters in our sample show a lower environmental impact than many pescatarians or vegetarians as depicted in Figure 2.

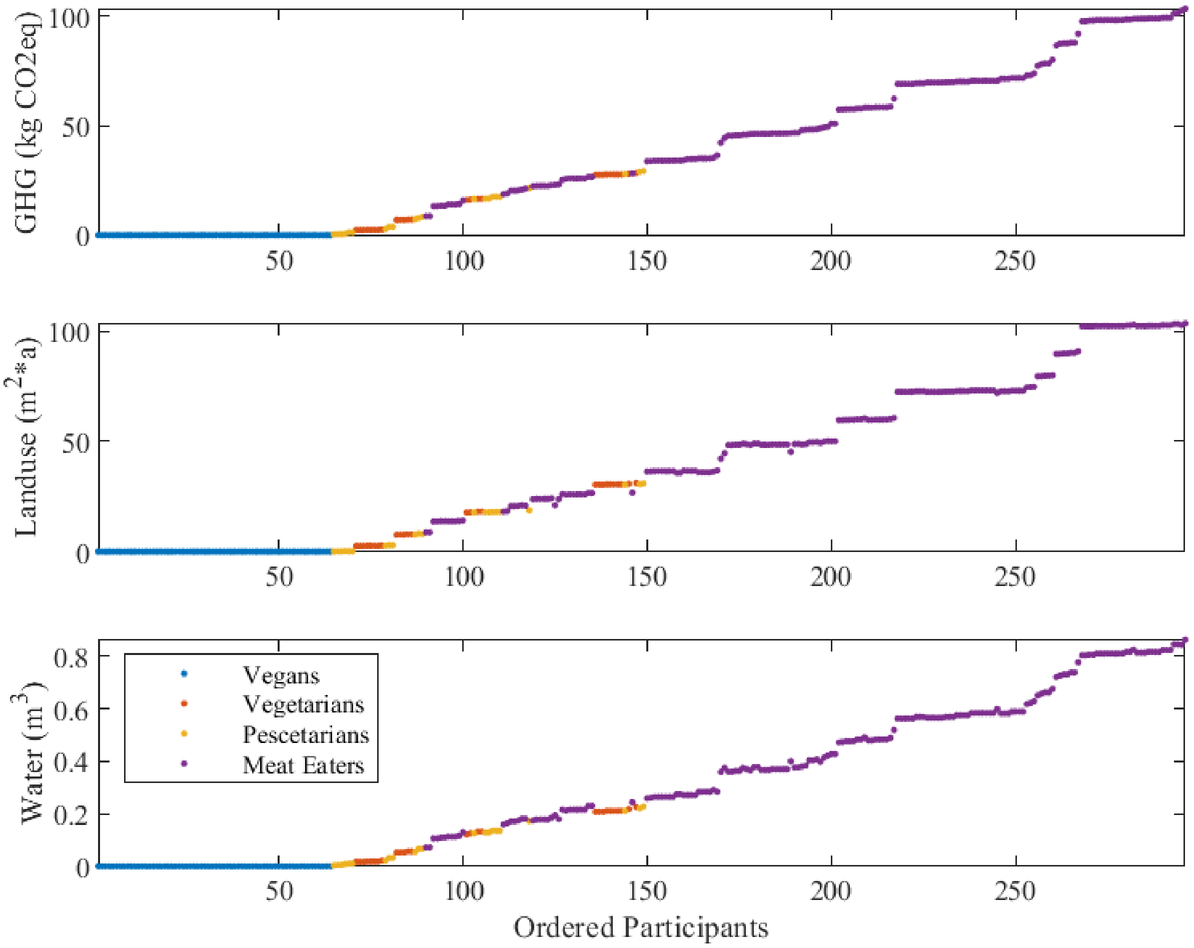

Figure 1: Environmental impact (GHG emissions, land use, water use) associated with individual diets in our sample of 307 respondents. The figure also shows that we gain much insight into individual dietary behavior by determining frequencies rather than working with respondents' diet types.

2 APPENDIX B - Full descriptive statistics and variable correlations

Descriptive Statistics

	N	Minimu	Maximu	Mean	Std.	Varianc	Skewness		Kurtosis	
	Statisti	Statistic	Statistic	Statistic	Statistic	Statistic	Statisti	Std.	Statisti	Std.
GHG emissions from	297	.00	103.51	37.3634	33.26153	1106.32	.468	.141	-1.046	.282
Land use from	297	.00	103.59	38.7752	34.28392	1175.38	.455	.141	-1.042	.282
Water from animal-	297	.00	.86	.3069	.27448	.075	.489	.141	-1.030	.282
Personal health	186	.00	100.00	21.7419	20.10185	404.084	1.086	.178	1.435	.355
Animal welfare	186	.00	100.00	44.5699	26.71892	713.900	.361	.178	-.651	.355
Environment and climate	186	.00	80.00	25.8871	18.44622	340.263	.664	.178	.364	.355
Weight control	186	.00	50.00	5.3817	10.48755	109.989	2.372	.178	5.687	.355
Intrinsic motivation	274	1.00	7.00	4.5730	1.41232	1.995	-.342	.147	-.343	.293
Integrated motivation	271	1.00	7.00	4.9271	1.57159	2.470	-.442	.148	-.690	.295
Identified motivation	276	3.00	7.00	5.8877	.93448	.873	-.722	.147	-.113	.292
Introjected	276	1.00	7.00	4.8234	1.39929	1.958	-.480	.147	-.293	.292
External motivation	273	1.00	5.75	1.8974	1.07999	1.166	1.424	.147	1.545	.294
Amotivation score	275	1.00	7.00	2.1655	1.19185	1.421	1.033	.147	.569	.293
Happiness Scale	299	.75	6.75	4.2408	1.46209	2.138	-.284	.141	-.667	.281
Connectedness with	299	1.57	5.00	3.8304	.71457	.511	-.484	.141	-.319	.281
Mindfulness score	299	2.41	5.54	3.9024	.58775	.345	.375	.141	-.355	.281
Meat reduction	300	0	2	1.37	.491	.241	.441	.141	-1.590	.281
Valid N (listwise)	165									

Correlations (Hypothesis 1)

		Happiness Scale	Connectedness	Mindfulness score
Happiness Scale score	Pearson Correlation	1	.355**	.629**
	Sig. (2-tailed)		.000	.000
	N	299	298	298
Connectedness with Nature score	Pearson Correlation	.355**	1	.398**
	Sig. (2-tailed)	.000		.000
	N	298	299	298
Mindfulness score	Pearson Correlation	.629**	.398**	1
	Sig. (2-tailed)	.000	.000	
	N	298	298	299

**. Correlation is significant at the 0.01 level (2-tailed).

Correlations (Hypothesis 2)

		Intrinsic	Integrated	Identified	Introjected	External	Amotivatio
Intrinsic motivation score	Pearson	1	.548**	.373**	.296**	-.012	-.157**
	Sig. (2-tailed)		.000	.000	.000	.845	.010
	N	274	267	273	273	269	272
Integrated motivation score	Pearson	.548**	1	.556**	.434**	-.131*	-.334**
	Sig. (2-tailed)	.000		.000	.000	.033	.000
	N	267	271	269	270	267	268
Identified motivation score	Pearson	.373**	.556**	1	.524**	-.287**	-.404**
	Sig. (2-tailed)	.000	.000		.000	.000	.000
	N	273	269	276	275	271	274

Introjected motivation score	Pearson	.296**	.434**	.524**	1	.061	-.151*
	Sig. (2-tailed)	.000	.000	.000		.314	.012
	N	273	270	275	276	271	274
External motivation score	Pearson	-.012	-.131*	-.287**	.061	1	.505**
	Sig. (2-tailed)	.845	.033	.000	.314		.000
	N	269	267	271	271	273	270
Amotivation score	Pearson	-.157**	-.334**	-.404**	-.151*	.505**	1
	Sig. (2-tailed)	.010	.000	.000	.012	.000	
	N	272	268	274	274	270	275

**. Correlation is significant at the 0.01 level (2-tailed).

*. Correlation is significant at the 0.05 level (2-tailed).

Correlations (Hypothesis 3, motivations for meat reduction)

		Personal health	Animal welfare	Environment and climate	Weight control
Personal health	Pearson Correlation	1	-.655**	-.265**	.253**
	Sig. (2-tailed)		.000	.000	.000
	N	186	186	186	186
Animal welfare	Pearson Correlation	-.655**	1	-.348**	-.459**
	Sig. (2-tailed)	.000		.000	.000
	N	186	186	186	186

Environment and climate	Pearson Correlation	-.265**	-.348**	1	-.235**
	Sig. (2-tailed)	.000	.000		.001
	N	186	186	186	186
Weight control	Pearson Correlation	.253**	-.459**	-.235**	1
	Sig. (2-tailed)	.000	.000	.001	
	N	186	186	186	186

**. Correlation is significant at the 0.01 level (2-tailed).

Correlations (Hypothesis 3, environmental impact from animal-protein consumption)

		GHG emissions	Land use from	Water from
GHG emissions from animal-protein	Pearson Correlation	1	.997**	.996**
	Sig. (2-tailed)		.000	.000
	N	297	297	297
Land use from animal-protein	Pearson Correlation	.997**	1	.999**
	Sig. (2-tailed)	.000		.000
	N	297	297	297
Water from animal-protein	Pearson Correlation	.996**	.999**	1
	Sig. (2-tailed)	.000	.000	
	N	297	297	297

**. Correlation is significant at the 0.01 level (2-tailed).

3 APPENDIX C – Hypothesis 2: non-significant results

The following motivation types were found to not show any significant differences between the three meditator groups: intrinsic motivation, identified motivation, introjected motivation and external motivation. The results are displayed in the following table.

Table 1: Games-Howell comparisons of four types of motivation towards the environment with non-significant results, including confidence intervals.

INTRINSIC MOTIVATION						
				Games-Howell Comparisons (p-value) [CI for the difference]		
Meditation Practice	*n*	Mean	*SD*	1	2	3
1. Non-practitioner	102	4.52	1.42			
2. Infrequent/ Novice practitioner	73	4.63	1.45	.870 [-.406 - .626]		
3. Advanced practitioner	81	4.53	1.44	.999 [-.509 - .494]	.899 [-.447 - .651]	
**IDENTIFIED MOTIVATION**						
				Games-Howell Comparisons (p-value) [CI for the difference]		
Meditation Practice	*n*	Mean	*SD*	1	2	3
1. Non-practitioner	102	5.88	.90			
2. Infrequent/ Novice practitioner	73	5.92	1.00	.956 [-.303 - .387]		
3. Advanced practitioner	81	5.93	.87	.901 [-.253 - .367]	.994 [-.3420 - .3728]	
**INTROJECTED MOTIVATION**						
				Games-Howell Comparisons (p-value) [CI for the difference]		
Meditation Practice	*n*	Mean	*SD*	1	2	3
1. Non-practitioner	102	4.92	1.42			
2. Infrequent/ Novice practitioner	73	5.00	1.36	.924 [-.576 - .417]		
3. Advanced practitioner	81	4.50	1.37	.105 [-.067 - .908]	.060 [-.017 – 1.017]	
**EXTERNAL MOTIVATION**						
				Games-Howell Comparisons (p-value) [CI for the difference]		
Meditation Practice	*n*	Mean	*SD*	1	2	3
1. Non-practitioner	102	2.00	1.19			
2. Infrequent/ Novice practitioner	73	1.98	1.03	.996 [-.380 - .408]		
3. Advanced practitioner	81	1.70	.93	.148 [-.076 - .664]	.181 -.093 - .652]	

4 APPENDIX D – Hypothesis 3: meat reduction status (gender differences)

When analyzing the reduction status by gender, the distribution patterns change (Figure 2 and 3). In all practitioner groups, more females report reductions in their meat consumptions than non-reductions and group differences were not statistically significant χ^2 (2, N = 186) = 4.85, p = .088.

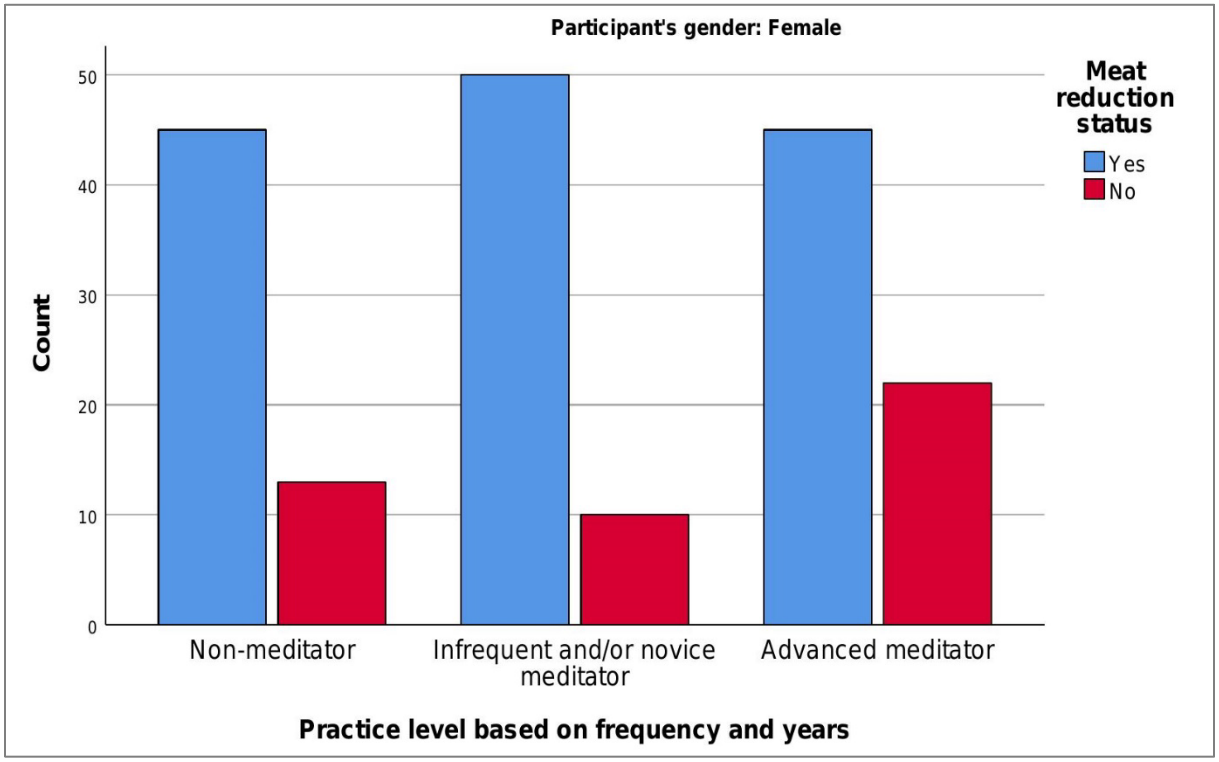

Figure 2. Animal-protein reduction statements of women: reduction levels are evenly distributed throughout the groups.

When observing male practitioner groups, more men state to not reduce their meat consumption in the non-practitioner group (47 non-reducers, 74.6%; 16 reducers, 25.4%). The proportion of non-reducers decreases slightly in the group of infrequent/novice practitioners (14 non-reducers, 63.6%; 8 reducers, 36.4%). However, in the advanced meditator group, more males report intentions to reduce meat consumption (5 non-reducers, 20%; 15 reducers, 80%). Group differences were statistically significant χ^2 (2, N = 110) = 22.39, p < .001.

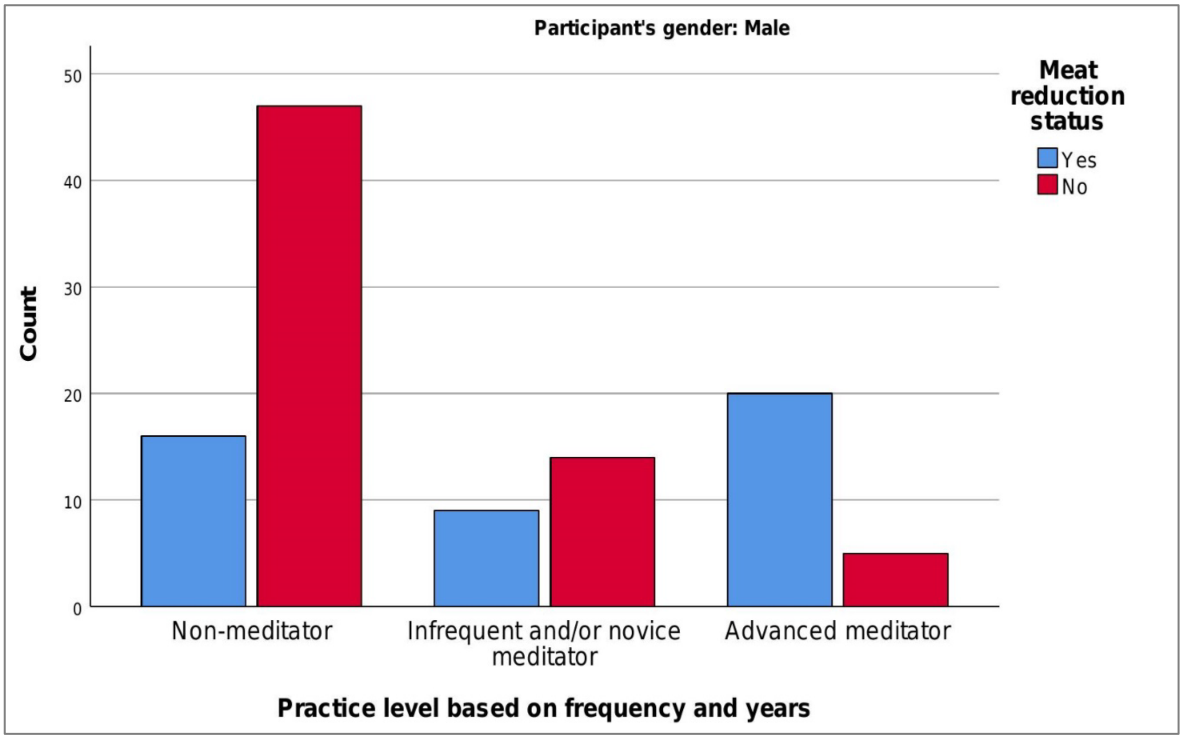

Figure 3 Animal-protein reduction statements of men: reduction levels increase with growing meditation experience.

5 APPENDIX E – Hypothesis 3: environmental impact (vegans excluded)

On the pages below we present excerpts from the SPSS script of the ANOVA on the environmental impact GHG emissions using the sample without Vegans. The groups result more clearly separated from each other and differences between all groups are significant. Respondents with advanced meditation experience emit significantly less GHG emissions linked to their animal-protein consumption.

The other environmental impacts (land occupation and water use) repeat the same pattern. To gain access to the rest of the script on the other indicators, please email the first author ute.thiermann15@imperial.ac.uk.

Between-Subjects Factors

		Value Label	N
Practice level based on frequency and years	.00	Non-meditator	95
	1.00	Infrequent and/or novice meditator	56
	2.00	Advanced meditator	80

Descriptive Statistics

Dependent Variable: GHG emissions from animal-protein

Practice level based on frequency and years	Mean	Std. Deviation	N
Non-meditator	59.7620	30.45141	95
Infrequent and/or novice meditator	46.7159	28.69746	56
Advanced meditator	33.7571	25.55738	80
Total	47.5933	30.46643	231

Levene's Test of Equality of Error Variances[a]

Dependent Variable: GHG emissions from animal-protein

F	df1	df2	Sig.
.980	2	228	.377

Tests the null hypothesis that the error variance of the dependent variable is equal across groups.

a. Design: Intercept + Gender_num + Practice_level

Tests of Between-Subjects Effects

Dependent Variable: GHG emissions from animal-protein

Source	Type III Sum of Squares	df	Mean Square	F	Sig.	Partial Eta Squared	Noncent. Parameter	Observed Power[b]
Corrected Model	38040.077[a]	3	12680.026	16.406	.000	.178	49.218	1.000
Intercept	17686.645	1	17686.645	22.884	.000	.092	22.884	.997
Gender_num	8614.447	1	8614.447	11.146	.001	.047	11.146	.914
Practice_level	17576.629	2	8788.314	11.371	.000	.091	22.741	.993
Error	175446.746	227	772.893					
Total	736729.834	231						

Corrected Total	213486.823	230						

a. R Squared = .178 (Adjusted R Squared = .167)

b. Computed using alpha = .05

Estimates

Dependent Variable: GHG emissions from animal-protein

Practice level based on frequency and years	Mean	Std. Error	95% Confidence Interval Lower Bound	95% Confidence Interval Upper Bound
Non-meditator	57.266[a]	2.949	51.456	63.076
Infrequent and/or novice meditator	47.694[a]	3.727	40.351	55.037
Advanced meditator	36.036[a]	3.182	29.766	42.307

a. Covariates appearing in the model are evaluated at the following values: Gender = 1.45.

Pairwise Comparisons

Dependent Variable: GHG emissions from animal-protein

(I) Practice level based on frequency and years	(J) Practice level based on frequency and years	Mean Difference (I-J)	Std. Error	Sig.[b]	95% Confidence Interval for Difference[b] Lower Bound	95% Confidence Interval for Difference[b] Upper Bound
Non-meditator	Infrequent and/or novice meditator	9.571*	4.798	.047	.117	19.026

	Advanced meditator	21.229*	4.455	.000	12.452	30.007
Infrequent and/or novice meditator	Non-meditator	-9.571*	4.798	.047	-19.026	-.117
	Advanced meditator	11.658*	4.859	.017	2.082	21.233
Advanced meditator	Non-meditator	-21.229*	4.455	.000	-30.007	-12.452
	Infrequent and/or novice meditator	-11.658*	4.859	.017	-21.233	-2.082

Based on estimated marginal means

*. The mean difference is significant at the .05 level.

b. Adjustment for multiple comparisons: Least Significant Difference (equivalent to no adjustments).
